# Prognostic Value of Hemoglobin, Albumin, Lymphocyte, Platelet (HALP) Score in Patients with Metastatic Renal Cell Carcinoma Treated with Nivolumab †

**DOI:** 10.3390/biomedicines13020484

**Published:** 2025-02-16

**Authors:** Zuzana Tomčová, Jana Obertová, Michal Chovanec, Zuzana Syčová-Milá, Katarína Štefániková, Eva Šlachtová, Monika Žák, Alexander Savka, Matej Hrnčár, Katarína Rejleková, Patrik Palacka

**Affiliations:** 1 2nd Department of Oncology, Faculty of Medicine, Comenius University and National Cancer Institute, Klenová 1, 833 10 Bratislava, Slovakia; 2 Department of Oncology, University General Hospital, Špitálska 6, 950 01 Nitra, Slovakia; 3 Cancer Research Institute, Biomedical Research Center, Slovak Academy of Sciences, Dúbravská cesta 9, 814 39 Bratislava, Slovakia; 4 Department of Oncology, East Slovak Cancer Institute, Rastislavova 43, 041 91 Košice, Slovakia; 5 Department of Oncology, F. D. Roosevelt University General Hospital, Nám. Ľ. Svobodu 1, 975 17 Banská Bystrica, Slovakia

**Keywords:** blood counts, albumin, renal cell carcinoma, metastatic disease, nivolumab

## Abstract

**Background:** Immunotherapy based on checkpoint inhibition is widely used in the treatment of metastatic renal cell carcinoma (RCC); however, predictive and prognostic biomarkers are yet to be explored. The objective of this study was to evaluate the prognostic value of the hemoglobin, albumin, lymphocyte, platelet (HALP) score in metastatic RCC patients receiving nivolumab. **Methods:** We enrolled 149 individuals (including 38 females) with a median age of 62 years, who were treated with nivolumab (at a dosage of 240 mg biweekly or 480 mg every 28 days) following progression on at least one tyrosine kinase inhibitor (TKI) between 2016 and 2024. The study population was dichotomized by the median HALP score (27.53), which was calculated as hemoglobin (g/L) × albumin (g/L) × absolute lymphocyte count/platelets (g/L) at immunotherapy initiation. Progression-free survival (PFS) and overall survival (OS) were estimated using the Kaplan–Meier method, with differences analyzed via a log-rank test. A multivariate Cox proportional hazards model was utilized for evaluation of the prognostic value of performance status, lactate dehydrogenase (LDH) levels, and HALP score. **Results:** At a median follow-up of 31.1 months, 122 patients had progressed on nivolumab and 87 had died. Poor performance status was associated with significantly worse PFS and OS (HR 0.20 and 0.14, respectively). Survival was worse in individuals with an LDH level higher than 1.5 times the normal range compared to those with lower LDH values (HR 0.45 for PFS and HR 0.41 for OS). Patients with low HALP scores had shorter PFS (HR 0.69) and OS (HR 0.58) versus patients with high HALP scores. In the multivariate analysis, the independent prognostic value of the HALP index for OS was revealed in a metastatic clear-cell RCC (ccRCC) population. **Conclusions:** The HALP score determined before nivolumab initiation as the second or third line of treatment is an independent prognostic factor of OS in metastatic ccRCC patients. Prospective validation could lead to the incorporation of this index into prognostic models for patients with RCC.

## 1. Introduction

Renal cell carcinoma (RCC) is the most common type of kidney cancer, with a worldwide incidence of 434,419 new cases and a mortality of 155,705 deaths in 2022 [1]. RCCs represent a heterogeneous group of malignancies with various clinical features [2]. The latest World Health Organization (WHO) classification of urogenital tumors introduced a novel molecular-driven renal tumor classification, reflecting the results obtained since the introduction of global massive parallel sequencing [3]. However, distinguishing between conventional clear-cell RCC (ccRCC) and a heterogenous group of non-cc RCCs still remains significant in clinical practice because treatment decisions are made based on this distinction [4].

One of the advances in systemic therapy for metastatic RCC (mRCC) accomplished in recent years is immunotherapy based on the inhibition of immune checkpoint proteins, including programmed cell death-1 (PD-1), an inhibitory receptor expressed on activated T and B cells, monocytes and other immune cells [5]. Its ligands, PD-L1 and PD-L2, are expressed on tumor cells and inhibit the activation of T cells through binding to their PD-1 receptors, thereby enabling the cancer to escape immune attack [6]. Nivolumab is a fully human immunoglobulin G4 PD-1 antibody that inhibits the cellular immune response through the disruption of the interaction of PD-1 with the appropriate ligands [7]. This immunotherapy was approved for the treatment of advanced RCC based on a randomized, open-label, phase 3 study [8]. CheckMate 025 showed its efficiency over everolimus in a population pretreated with a maximum of two previous regimens of antiangiogenic therapy (pazopanib, sunitinib, or axitinib) [9]. Clinical outcomes associated with nivolumab have also been demonstrated in real-world scenarios [10,11].

The immune system can normally suppress or slow tumor growth through the recognition and removal of tumor cells. However, in the context of chronic inflammation, inflammatory cells and cytokines affect the proliferation, survival, and invasion of tumor cells and angiogenesis by acting as promoters of tumorigenesis [12]. The inflammatory indices calculated based on pretreatment counts of neutrophils, lymphocytes, and platelets are associated with OS in patients with multiple malignancies treated with various therapies, including nivolumab for mRCC [13].

Anemia (defined as hemoglobin levels less than the lower limit of the normal range) and thrombocytosis (defined as platelet counts higher than the upper limit of the normal range), as well-recognized negative prognostic factors, are included in risk models that are broadly used for mRCC. The Memorial Sloan Kettering Cancer Center (MSKCC) integrates factors such as time from diagnosis to systemic treatment of less than 1 year, anemia, calcium level greater than 10 mg/dL, and an LDH level greater than 1.5× the upper limit of the normal range and a performance status of <80% (Karnofsky) [14]; meanwhile, the International mRCC Database Consortium (IMDC) operates with factors such as less than 1 year from time of diagnosis to the initiation of systemic therapy, a Karnofsky performance status of <80%, anemia, neutrophils greater than the upper limit of the normal range, and thrombocytosis [15]. In the era of tyrosine kinase inhibitors (TKIs), a meta-analysis has demonstrated the role of pre-treatment with albumin in predicting the prognosis of mRCC patients. A lower serum albumin level is significantly associated with worse progression-free survival (PFS) and OS [16].

The HALP score combines hemoglobin and albumin levels with lymphocyte and platelet counts. It was introduced by Chen et al. as a novel immune–nutritional prognostic biomarker in patients with gastric carcinoma following surgery in 2015 [17], and subsequently studied in several malignancies with conflicting results [18]. Nevertheless, a meta-analysis has revealed that a low HALP score is associated with worse OS, cancer-specific survival (CSS), and PFS/disease-free survival (DFS)/relapse-free survival (RFS) across various tumor types and stages [19]. Some studies have also shown an association between the index and response to immunotherapy, as well as chemotherapy [18].

However, reports on the impact of HALP score on mRCC are limited. If the index has a clinical impact on immunotherapy, we hypothesized that this could be a promising, easy-to-use biomarker for use in the management of mRCC. To test this hypothesis, we evaluated the potential associations of the HALP score with clinical outcomes in mRCC patients who received nivolumab in the second or third line of treatment, following progression on at least one TKI.

## 2. Patients and Methods

### 2.1. Selection of Patients

All enrolled individuals were treated with immunotherapy at one of the following institutions: National Cancer Institute, Bratislava, Slovakia (NCI); East Slovak Cancer Institute, Košice, Slovakia (ESCI); F. D. Roosevelt University General Hospital, Banská Bystrica, Slovakia (RUGH); and University General Hospital, Nitra, Slovakia (UGH). The selection of patients was extracted from the local databases by the investigators P.P., J.O., M.C., Z.S., and K.R. at the NCI; K.S., E.S., and M.Z. at the ESCI; A.S. and M.H. at the RUGH; and Z.T. at the UGH.

Eligible patients were those older than 18 years with histologically confirmed mRCC. The disease was radiologically documented and measurable, according to the Response Evaluation Criteria in Solid Tumors (RECIST version 1.1) [20]. All patients had been pretreated with one or two lines of previous systemic therapy. Sunitinib and pazopanib were among the permitted TKIs. Axitinib is not reimbursed and, hence, not prescribed by medical oncologists in Slovakia. Everolimus as second-line treatment was allowed, due to national restrictions related to immunotherapy in mRCC for a certain time.

Exclusion criteria comprised a known acute viral or bacterial infection, use of corticosteroids or other immunosuppressive medication, parenteral nutrition or nutritional supplements, and a blood transfusion within 4 weeks prior to the first application of nivolumab. Patients with an active secondary malignancy or a known hematological or rheumatological disease were also excluded from the analysis. On the other hand, history of current central nervous system (CNS) metastases was allowed, when stable.

### 2.2. Study Design

**Figure 1:**
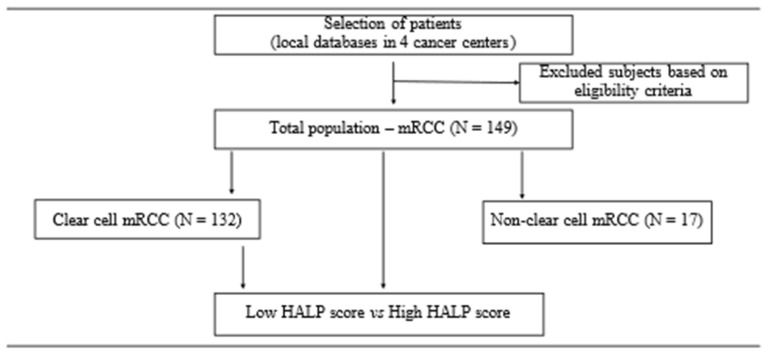
Study design flowchart. The local databases of four cancer centers in Slovakia were used for selection of patients with mRCC. The total study population consisted of patients with mRCC, regardless of histology, who met the inclusion and exclusion criteria. The total population and subpopulation with solely clear-cell mRCC were divided into subgroups based on the median HALP score (27.53). Abbreviations: mRCC, metastatic renal cell carcinoma; N, number of patients; HALP, a score combining hemoglobin and albumin levels with lymphocyte and platelet counts.

This was a retrospective analysis reflecting real-world practice, which was approved by the independent Ethics Committee at the NCI and conducted in accordance with Good Clinical Practice guidelines defined by the International Conference on Harmonisation. The HALP score was calculated as hemoglobin (G/l) × albumin (G/l) × an absolute lymphocyte count/platelet count (G/l). A median HALP score of 27.3 (3.28–27.53) was determined in the total population. Based on the median HALP, the total population and subsequently the subpopulation of clear-cell mRCC were divided into two subgroups: low HALP score (≤27.53) and high HALP score (>27.53). The study design flowchart is shown in Figure 1.

The risk factors involved in the MSKCC model (hypercalcemia, LDH more than 1.5-times the upper limit, and poor performance status 2 by Eastern Cooperative Oncology Group; but without anemia, which was factored into the HALP), number of previous therapy lines (one or two) for mRCC, time from previous line start to nivolumab initiation less than 12 months, histology (ccRCC vs. non-ccRCC), and the median HALP score were selected as the candidate factors for survival analyses in both populations (total and clear-cell mRCC).

### 2.3. Treatment and End Points

According to local practice, all patients received 240 mg nivolumab every 2 weeks or 480 mg nivolumab every 4 weeks as a 60-min intravenous infusion. Treatment was continued until disease progression (clinical progression or progression confirmed by a radiologist), the occurrence of unacceptable toxicity, the patient’s request to terminate therapy, or death. All patients who received one or more doses of nivolumab were included in the efficacy analysis. Herein, the occurrence of adverse events was not evaluated.

PFS was defined as the time from nivolumab initiation to disease progression, last follow-up, or death from any cause. OS was defined as the time from nivolumab initiation to last follow-up or death from any cause. The ORR was defined as the number of individuals with a complete response or a partial response divided by the number of patients who initiated immunotherapy. The best overall response was defined as the best response from the time of nivolumab initiation to disease progression or subsequent treatment. To assess the tumor response, imaging data were independently evaluated by both a radiologist and an investigator.

### 2.4. Statistical Analyses

The normality of numerical data distribution was assessed using the Kolmogorov–Smirnov test. The normally distributed numerical variables are expressed as means and standard deviations (SD) and were compared between groups using the *t*-test or one-way analysis of variance (ANOVA). Numerical variables with non-normal distribution are expressed as the median (interquartile range (IQR)) and were compared between groups using the non-parametric Kruskal–Wallis ANOVA and Mann–Whitney U-test. The categorical variables are presented as relative and absolute frequencies and were compared using the Fisher’s exact test or the Chi-square test, if appropriate.

PFS and OS were estimated using the Kaplan–Meier method and differences were assessed using the log-rank test. Cox regression analysis was used to explore multivariate associations with survival. To investigate the predictors of response to treatment, univariate and multivariate logistic regression analyses were performed. A value of *p* < 0.05 was considered significant. The statistical analyses were performed with NCSS 2024 version 24.0.3 (Kaysville, UT, USA).

## 3. Results

### 3.1. Characteristics of Patients, Tumor, Disease and Treatments

The dates of nivolumab initiation for the first and last patients were 13 January 2016 and 8 November 2023, respectively. The data cut-off for this analysis was 29 January 2024.

For clinical efficacy, a total of 149 patients who received immunotherapy were analyzed. The majority were males (74.5%) with ccRCC, including its variants (88.6%). Performance status according to Eastern Cooperative Oncology Group (ECOG) scores of 0, 1 and 2 was 31.5, 50.3 and 18.2%, respectively. In the first line, patients had failed sunitinib in 79.2% and pazopanib in 20.8%. At the time of nivolumab initiation, common sites of distant metastases were lungs (73.8%), lymph nodes (69.8%) and bones (39.6%). Nivolumab was received as second line in 60.4% and 39.6% in third line. The median of immunotherapy doses was 12 (1–116).

The median duration of follow-up for the study population was 31.1 (27.8–37.1) months. Complete response was achieved in 2.7% and partial response in 28.9%. Therefore, the ORR was 31.5%. All essential characteristics of the study population are detailed in Table 1.

### 3.2. Total Population

**Figure 2:**
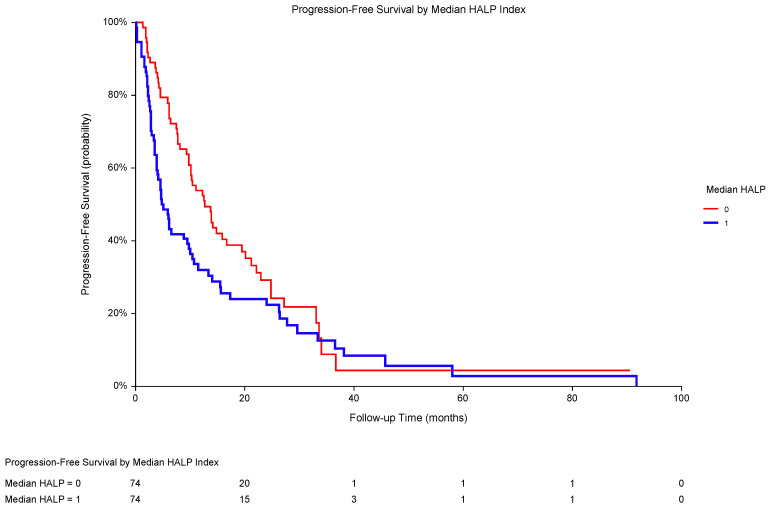
In metastatic RCC patients (the total population), PFS was significantly worse with a low HALP score (1; median PFS 4.8 months) compared to that with a high HALP score (0; median PFS 12.7 months) (*p* = 0.0382; HR 0.69, 95% CI 0.48–0.99). The median HALP score was 27.53, and HALP ≤ 27.53 was considered low. Abbreviations: RCC, renal cell carcinoma; PFS, progression-free survival; HALP, a score combining pretreatment hemoglobin, albumin, lymphocytes, and platelets; HR, hazard ratio; CI, confidence interval.

Considering an identical approach to the treatment of individuals with metastatic ccRCC and non-ccRCC in the study population, we performed univariate analyses of survival according to all selected potential risk factors. Of them, poor performance status (ECOG 2), high serum LDH, and low HALP score were identified as significant predictors of PFS (Table 2). PFS was significantly shorter in patients with low HALP scores (median PFS 4.8 months) compared to that in individuals with high HALP scores (median PFS 12.7 months, *p* = 0.0382; HR 0.69, 95% CI 0.48–0.99; Figure 2). A multivariate analysis showed the independent prognostic value of performance status and LDH, but not HALP score (Table 3).

**Figure 3:**
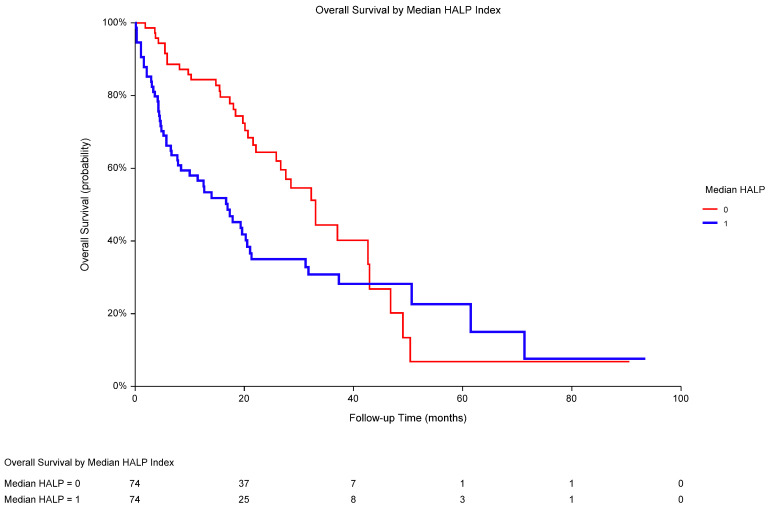
In metastatic RCC, overall survival was significantly worse in low-HALP-score patients (1; median OS 16.9 months) versus that in high-HALP-score patients (0; 33.0 months; *p* = 0.0108; HR 0.58, 95% CI 0.38–0.89). The median HALP score was 27.53, and HALP ≤ 27.53 was considered low. Abbreviations: RCC, renal cell carcinoma; OS, overall survival; HALP, a score combining pretreatment hemoglobin, albumin, lymphocytes, and platelets; HR, hazard ratio; CI, confidence interval.

In the univariate analyses, poor performance status, high LDH, and low HALP score were recognized as significant predictors of OS (Table 4). OS was significantly worse in low-HALP individuals when compared to that in high-HALP patients (median OS 16.9 vs. 33.0 months; *p* = 0.0108; HR 0.58, 95% CI 0.38–0.89; Figure 3). A multivariate analysis revealed the independent prognostic value of performance status and LDH but not HALP score in the total population (Table 5).

Using univariate logistic regression analysis, we did not observe the HALP score as a predictor of the response to nivolumab (30.7% ORR in low-HALP-score patients vs. 32.4% in high-HALP-score patients, *p* = 0.4570).

### 3.3. Metastatic ccRCC Population

**Figure 4:**
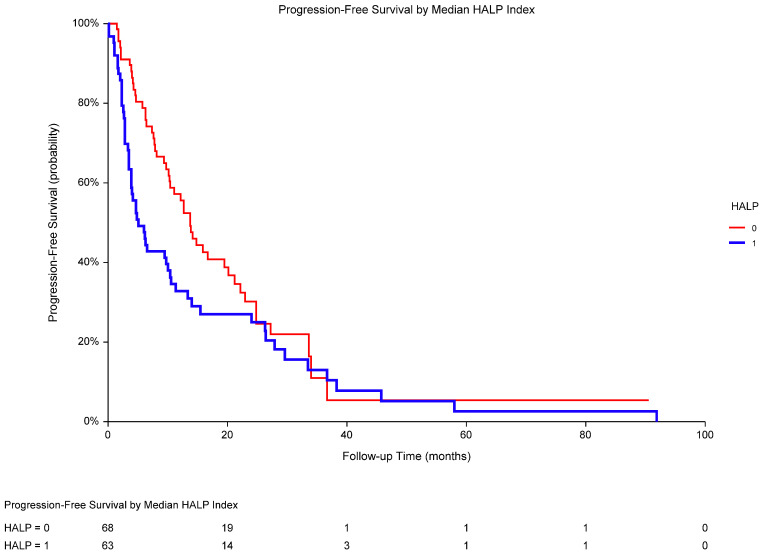
In metastatic clear-cell RCC, PFS in low-HALP-score patients was significantly worse (1; median PFS 5.1 months) when compared to that in high-HALP-score patients (0; median PFS 13.8 months; *p* = 0.0432; HR 0.68, 95% CI 0.46–1.00). Median HALP score was 27.53, HALP ≤ 27.53 was considered low. Abbreviations: RCC, renal cell carcinoma; PFS, progression-free survival; HALP, a score combining pretreatment hemoglobin, albumin, lymphocytes, and platelets; HR, hazard ratio; CI, confidence interval.

Regarding different prognoses, we also performed univariate analyses of survival according to all selected potential risk factors in the metastatic ccRCC population (N = 132). Pretreatment performance status with respect to ECOG, serum LDH, and HALP score were identified as the predictors of PFS (Table 6). In particular, PFS was significantly worse in low-HALP-score patients (median PFS 5.1 months) when compared to that in high-HALP-score patients (median PFS 13.8 months; *p* = 0.0432; HR 0.68, 95% CI 0.46–1.00; Figure 4). A multivariate analysis demonstrated the independent prognostic value of performance status and LDH but not HALP score in the metastatic ccRCC population (Table 7).

**Figure 5:**
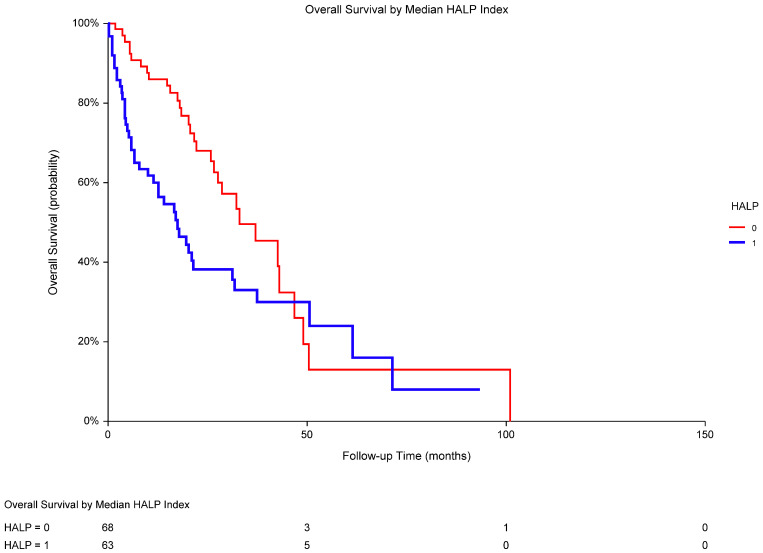
In metastatic clear-cell RCC, OS was significantly worse in patients with low-HALP-score (1; median OS 17.4 months) when compared to that in high-HALP-score patients (0; median OS 33.0 months; *p* = 0.0114; HR 0.56, 95% CI 0.35–0.89). The median HALP score was 27.53, and HALP ≤ 27.53 was considered low. Abbreviations: RCC, renal cell carcinoma; OS, overall survival; HALP, a score combining pretreatment hemoglobin, albumin, lymphocytes, and platelets; HR, hazard ratio; CI, confidence interval.

In the univariate analyses, performance status, LDH, and HALP score were identified as predictors of OS in the metastatic ccRCC population (Table 8). OS was significantly worse in low HALP individuals, compared to high-HALP individuals (median OS 17.4 vs. 33.0 months; *p* = 0.0114; HR 0.56, 95% CI 0.35–0.89; Figure 5). A multivariate analysis revealed the independent prognostic value of performance status, LDH and HALP score in the metastatic ccRCC population (Table 9).

Pretreatment HALP score did not predict the response to nivolumab in the metastatic ccRCC population (30.9% ORR in low-HALP-score patients vs. 31.3% in high-HALP-score patients, *p* = 0.3301).

## 4. Discussion

We performed a retrospective analysis to explore the possible associations between the immune–nutritional HALP score (combining pretreatment hemoglobin, serum albumin, lymphocyte count, and platelets) with clinical outcomes in mRCC patients who received the anti-PD-1 monoclonal antibody nivolumab following progression on a maximum of two previous lines of systemic therapy (including at least one TKI). The enrolled patients came from four cancer centers in Slovakia. The independent prognostic value of HALP score along with serum LDH and performance status was evaluated in a metastatic ccRCC population.

Metastatic RCC is a fascinating malignancy with a dramatically changing treatment landscape in recent years. Since the beginning of this century, cytokines have gradually been replaced by tyrosine kinase inhibitors (TKIs), inhibitors of the mammalian target of rapamycin (mTOR) signaling pathway, second-generation immunotherapy based on the inhibition of immune checkpoints and, finally, an inhibitor of hypoxia-inducible factor (HIF)-1 regulatory pathway have received attention in the field. Following the publication of a phase 3 study comparing immunotherapy to everolimus [8], the US Food and Drug Administration and European Medicines Agency approved nivolumab after progression on at least one TKI in metastatic ccRCC. Patients with non-ccRCC were not included in this study. Nevertheless, despite the absence of prospective data, nivolumab has also been used in this population [21,22,23].

Cancer cell proliferation and migration, angiogenesis, and tumor spread are mediated by inflammation in the context of RCC. These alterations in the immune cells lead to immunosuppression and immune cell infiltration [24]. Testing immune–inflammatory circulating cells (neutrophils, lymphocytes, and platelets) in blood and quantifying the levels of inflammation in terms of various indices, including the neutrophil-to-lymphocyte ratio (NLR), platelet-to-lymphocyte ratio (PLR), and systemic immune–inflammation index (SII), may have prognostic value [13,25,26,27].

Hypoalbuminemia is associated with decreased OS and CSS, and an enhanced risk of all-cause and disease-specific early mortality in individuals with mRCC undergoing cytoreductive nephrectomy [28]. However, serum albumin may not indicate the true nutritional status and has certain limitations as a predictor of survival. Its prognostic value can be questioned under circumstances influenced by factors such as diet, overhydration, or liver diseases. In the 1980s, Onodera et al. developed a prognostic nutritional index (PNI) based on serum albumin level and total lymphocyte count in peripheral blood. When validated prospectively, the PNI provided an accurate quantitative estimate of operative risk for malnourished cancer patients undergoing gastrointestinal surgery [29]. The PNI has shown certain prognostic value, even in individuals with RCC [30,31,32,33].

Unlike the previous indices, the HALP score may be used to assess the immune status and nutritional status at the same time. Its independent prognostic role was first described in the context of gastric cancer. Patients with a high HALP score had significantly better prognosis than those with a low HALP score [17]. These results led to research from early to advanced stages of malignancies including non-small cell lung cancer patients receiving conventional platinum-based chemotherapy. In a retrospective analysis, an optimal cut-off for the HALP score was identified as 28.02. As low HALP score, male sex, pathological types other than adenocarcinoma and progression on chemotherapy were independently associated with worse survival, the authors established a nomogram based on these factors with a better predictive capability for OS than a separate HALP score [34].

Peng et al. revealed the HALP score as an independent predictor of CSS in a cohort of 1360 patients who underwent a nephrectomy due to ccRCC and non-ccRCC. Non-ccRCC represented 10% of the study population, and the HALP score cut-off value was 31.2. The data were analyzed at a median follow-up of 67 months. During follow-up, 10.2% of patients had died due to RCC and the 5-year estimated CSS was 89.4%. The risk model, which included the TNM stage, the Fuhrman grade, and the HALP score, had better prognostic predictive accuracy than the system solely based on TNM [35].

In a cohort of patients with mRCC, Ekinci et al. determined a cut-off value for the HALP score of 0.277 through calculating sensitivity and specificity values based on OS and PFS and examining the area under the receiver operating characteristic curve (ROC). The study population consisted of ccRCC (79.7%) and non-ccRRC (20.3%) patients. The HALP score was strongly associated with OS (mOS for low- and high-HALP subgroups were 17.7 and 89.7 months, respectively). In the multivariate analysis, the HALP score was an independent prognostic factor [36]. However, from this study, it is not obvious whether the patients received any systemic therapies, their type, and the number of lines. These data are significant as available treatments and their sequential use affect OS.

The only published data on the association between the HALP score and selected clinical outcomes in mRCC patients, who received nivolumab as second line following progression on TKIs (pazopanib or sunitinib), come from a small single-center retrospective study of 45 subjects [37]. The HALP cut-off value was found to be 16.98. Considering that this study reported five patients having mRCC with sarcomatoid features but no non-ccRCC, we assume that the study population consisted only of clear-cell histology, which corresponds with the metastatic ccRCC population in our study. PFS (stated as a mean) was significantly two-fold better in individuals with a high HALP score compared to that in those with a low HALP score (12.0 months vs. 6.0 months). They also found significant correlations between PFS and serum albumin, but not LDH, performance status, best radiological response, number of metastasis sites, and metastasis sites. The associations between HALP score and OS were not published [37].

To the best of our knowledge, our multicentric retrospective analysis is the first study revealing a significant association between HALP score and OS in mRCC patients treated with immunotherapy. The total population, consisting of all patients who met the inclusion criteria regardless of histology, was dichotomized by a median HALP of 27.53. In the univariate analyses, patients with poor performance status (ECOG 2), high serum LDH (more than 1.5 times the upper limit) and low HALP score (≤27.53) had significantly worse survival in terms of PFS and OS than those with good performance status (ECOG 0–1), normal serum LDH (1.5 times the upper limit or lower) and high HALP score (>27.53). The multivariate analysis showed an independent prognostic value of performance status and LDH for PFS and OS. In a metastatic ccRCC population, poor performance status, high serum LDH, and low HALP score (≤27.53) were associated with shorter PFS and OS. The multivariate analysis revealed an independent prognostic role of performance status and LDH for PFS and performance status, LDH and HALP for OS. Neither in the total population nor in the metastatic ccRCC subgroup was a correlation between ORR and HALP score detected. Several studies and a meta-analysis [38] have reported an association between serum LDH and clinical outcomes in RCC, including metastatic stage. In another meta-analysis, poor performance status predicted unfavorable prognostic outcomes in mRCC patients receiving TKIs [39].

The major limitations of our study are its retrospective character and relatively small sample size, thus constraining the statistical power for some of the presented analyses. In addition, comorbid conditions and contemporaneous medications represent confounding factors with a potential influence on HALP score, despite our efforts to eliminate them by excluding such patients from the final analysis. The wider use of the HALP score in clinical practice with the aim of improving prognoses is also complicated, due to the various cut-off values determined within published papers. Moreover, its prospective validation in a larger sample size is needed.

To conclude, the HALP score, as an immune–nutritional index, has gained well-founded attention as a novel biomarker with the ability to predict survival in various malignancies. An undisputed advantage is its simplicity and practically zero cost, as all required parameters are examined as a standard approach for each patient before initiation of immunotherapy. Our study detected an association between low HALP and worse PFS and OS in both populations—metastatic RCC and metastatic ccRCC—treated with nivolumab as second or third line. The HALP score was shown to be an independent prognostic factor for metastatic ccRCC patients. However, for validation of these findings and to overcome the study’s limitations, further research is needed. Once validated in a prospective trial, the incorporation of the HALP score into prognostic models could be warranted.

## Figures and Tables

**Table 1:** Characteristics of patients, tumors, disease, and treatments: total population, subgroups with low and high HALP.

	Total		Low HALP		High HALP	
Characteristics	N	%	N	%	N	%
Age (years)						
Median (IQR)	62 (28–79)		62 (28–78)		63 (33–79)	
Sex						
Male	111	74.5	55	73.3	56	75.7
Female	38	25.5	20	26.7	18	24.3
Performance status (ECOG)						
0	47	31.5	19	25.3	28	37.8
1	75	50.3	33	44.0	42	56.8
2	27	18.2	23	30.7	4	5.4
Histology						
Clear-cell carcinoma *	132	88.6	64	85.3	68	91.9
Non-clear-cell carcinoma **	17	11.4	11	14.7	6	8.1
Cytoreductive nephrectomy						
Yes	124	83.2	58	77.3	66	89.2
No	25	16.8	17	22.7	8	10.8
Sites of distant metastasis ***						
Lymph node(s)	104	69.8	60	80.0	44	59.5
Bone(s)	59	39.6	35	46.7	24	32.4
Lungs	110	73.8	56	74.7	54	73.0
Liver	45	30.2	27	36.0	18	24.3
Pleura	31	20.8	23	30.7	8	10.8
Pancreas	12	8.1	7	9.3	5	6.8
Adrenal gland	40	26.9	21	28.0	19	25.7
CNS	8	5.4	2	2.7	6	8.1
Other sites	25	16.8	18	24.0	7	9.5
First-line treatment						
Sunitinib	118	79.2	60	80.0	57	77.0
Pazopanib	31	20.8	15	20.0	17	23.0
Second-line treatment						
Sunitinib	6	4.0	3	4.0	3	4.1
Pazopanib	15	10.1	6	8.0	9	12.2
Everolimus	38	25.5	25	33.3	13	17.5
Nivolumab	90	60.4	41	54.7	49	66.2
Third-line treatment						
Nivolumab	59	39.6	33	55.9	26	44.1
Number of doses ****						
Median number (IQR)	12 (1–116)		10 (1–116)		19 (1–75)	
Best response to nivolumab						
Complete response	4	2.7	2	2.7	2	2.7
Partial response	43	28.9	21	28.0	22	29.7
Stable disease	39	26.2	14	18.7	25	33.8
Progression	63	42.2	38	50.6	25	33.8

Abbreviations: IQR, interquartile range; N, number of patients; ECOG, Eastern Cooperative Oncology Group; CNS, central nervous system. * Twelve patients with variants (7 sarcomatoid variants and 5 rhabdoid variants) were included. ** Ten patients with papillary RCC (pRCC), 4 patients with not otherwise specified (NOS) RCC, and 3 patients with MiTF/TFE translocation RCC (tRCC). *** At the time of nivolumab initiation. **** Nivolumab 480 mg once a month or 2 applications of nivolumab 240 mg biweekly were considered for one dose.

**Table 2:** Progression-free survival by risk factors in the total population (univariate analyses).

	N	mPFS (95% CI) in Months	HR (95% CI)	*p*
ECOG 2 vs. ECOG 0–1	27 vs. 122	2.7 (1.6–3.5) vs. 12.7 (10.0–15.6)	0.20 (0.09–0.44)	<0.0001
Non-ccRCC yes vs. no	17 vs. 132	5.9 (3.0–9.7) vs. 10.3 (7.4–13.3)	0.68 (0.37–1.26)	0.1493
Hypercalcemia yes vs. no	8 vs. 141	4.0 (2.8–4.1) vs. 9.7 (6.5–12.2)	0.57 (0.21–1.53)	0.1414
High LDH * yes vs. no	19 vs. 130	2.8 (2.0–6.2) vs. 10.3 (7.8–13.8)	0.45 (0.22–0.93)	0.0018
HALP Low ** vs. high	75 vs. 74	4.8 (3.9–9.5) vs. 12.7 (9.7–16.7)	0.69 (0.48–0.99)	0.0382
PFS0 < 12 m. yes vs. no	99 vs. 50	8.8 (5.9–11.4) vs. 10.4 (7.4–15.6)	0.84 (0.58–1.22)	0.3707
Nivolumab third line yes vs. no	59 vs. 90	9.5 (5.8–12.6) vs. 10.1 (6.4–13.7)	0.91 (0.63–1.31)	0.5969

Abbreviations: N, number of patients; ECOG, Eastern Cooperative Oncology Group; ccRCC, clear-cell renal cell carcinoma; LDH, lactate dehydrogenase; HALP, a score combining pretreatment hemoglobin, albumin, lymphocytes, and platelets; PFS0, progression-free survival of previous line of treatment in months (m.); mPFS, median progression-free survival; CI, confidence interval; HR, hazard ratio; * more than 1.5 times the upper limit; ** HALP ≤ 27.53 was considered low.

**Table 3:** Progression-free survival by risk factors in the total population (multivariate analysis).

Variables	*p*
Poor performance status (ECOG 2)	<0.0001
High LDH (more than 1.5 times the upper limit)	0.0008
HALP ≤ 27.53	0.7422

Abbreviations: ECOG, Eastern Cooperative Oncology Group; LDH, lactate dehydrogenase; HALP, a score combining pretreatment hemoglobin, albumin, lymphocytes, and platelets.

**Table 4:** Overall survival by risk factors in the total population (univariate analyses).

	N	mOS (95% CI) in Months	HR (95% CI)	*p*
ECOG 2 vs. ECOG 0–1	27 vs. 122	4.3 (1.6–5.2) vs. 32.2 (25.9–42.7)	0.14 (0.05–0.35)	<0.0001
Non-ccRCC yes vs. no	17 vs. 132	15.5 (4.6–19.8) vs. 27.6 (20.2–33.0)	0.56 (0.27–1.17)	0.0515
Hypercalcemia yes vs. no	8 vs. 141	27.6 (4.4–31.2) vs. 22.1 (19.6–32.2)	0.64 (0.21–1.93)	0.3219
High LDH * yes vs. no	19 vs. 130	7.7 (3.7–19.6) vs. 28.6 (20.2–33.0)	0.41 (0.18–0.95)	0.0020
HALP Low ** vs. high	75 vs. 74	16.9 (7.9–20.6) vs. 33.0 (25.9–42.7)	0.58 (0.38–0.89)	0.0108
PFS0 < 12 m. yes vs. no	99 vs. 50	21.4 (16.9–28.6) vs. 32.2 (20.1–42.7)	0.80 (0.52–1.24)	0.3317
Nivolumab third line yes vs. no	59 vs. 90	18.0 (10.3–27.6) vs. 31.2 (20.6–42.7)	0.73 (0.47–1.12)	0.1333

Abbreviations: N, number of patients; ECOG, Eastern Cooperative Oncology Group; ccRCC, clear-cell renal cell carcinoma; LDH, lactate dehydrogenase; HALP, a score combining pretreatment hemoglobin, albumin, lymphocytes, and platelets; PFS0, progression-free survival of previous line of treatment in months (m.); mOS, median overall survival; CI, confidence interval; HR, hazard ratio; * more than 1.5 times the upper limit; ** HALP ≤ 27.53 was considered low.

**Table 5:** Overall survival by risk factors in the total population (multivariate analysis).

Variables	*p*
Poor performance status (ECOG 2)	<0.0001
High LDH (more than 1.5 times the upper limit)	0.0008
HALP ≤ 27.53	0.4775

Abbreviations: ECOG, Eastern Cooperative Oncology Group; LDH, lactate dehydrogenase; HALP, a score combining pretreatment hemoglobin, albumin, lymphocytes, and platelets.

**Table 6:** Progression-free survival by risk factors in the metastatic ccRCC population (univariate analyses).

	N	mOS (95% CI) in Months	HR (95% CI)	*p*
ECOG 2 vs. ECOG 0–1	24 vs. 108	2.8 (1.8–3.5) vs. 13.8 (10.4–16.7)	0.18 (0.07–0.44)	<0.0001
ccRCC variant yes vs. no	11 vs. 121	7.7 (4.8–13.8) vs. 10.3 (6.5–13.3)	1.01 (0.49–2.07)	0.9761
Hypercalcemia yes vs. no	8 vs. 124	4.0 (2.8–4.1) vs. 10.4 (7.6–13.7)	0.54 (0.20–1.48)	0.1084
High LDH * yes vs. no	12 vs. 120	2.8 (1.6–7.6) vs. 10.4 (7.8–13.9)	0.48 (0.19–1.23)	0.0281
HALP Low ** vs. high	64 vs. 68	5.1 (3.9–9.7) vs. 13.8 (10.3–19.5)	0.68 (0.46–1.00)	0.0432
PFS0 < 12 m. yes vs. no	86 vs. 46	10.0 (6.2–13.7) vs. 10.4 (7.4–15.5)	0.89 (0.60–1.32)	0.5592
Nivolumab third line yes vs. no	52 vs. 80	9.7 (6.0–14.1) vs. 10.4 (6.4–13.9)	0.94 (0.64–1.39)	0.7454

Abbreviations: N, number of patients; ECOG, Eastern Cooperative Oncology Group; ccRCC, clear-cell renal cell carcinoma; LDH, lactate dehydrogenase; HALP, a score combining pretreatment hemoglobin, albumin, lymphocytes, and platelets; PFS0, progression-free survival of previous line of treatment in months (m.); mPFS, median progression-free survival; CI, confidence interval; HR, hazard ratio; * more than 1.5 times the upper limit; ** HALP ≤ 27.53 was considered low.

**Table 7:** Progression-free survival by risk factors in the metastatic ccRCC population (multivariate analysis).

Variables	*p*
Poor performance status (ECOG 2)	<0.0001
High LDH (more than 1.5 times the upper limit)	0.0226
HALP ≤ 27.53	0.9155

Abbreviations: ECOG, Eastern Cooperative Oncology Group; LDH, lactate dehydrogenase; HALP, a score combining pretreatment hemoglobin, albumin, lymphocytes, and platelets.

**Table 8:** Overall survival by risk factors in the metastatic ccRCC population (univariate analyses).

	N	mOS (95% CI) in Months	HR (95% CI)	*p*
ECOG 2 vs. ECOG 0–1	24 vs. 108	4.3 (1.9–5.2) vs. 37.1 (27.6–42.9)	0.12 (0.04–0.34)	<0.0001
ccRCC variant yes vs. no	11 vs. 121	20.1 (6.6–28.6) vs. 31.2 (20.7–37.4)	0.74 (0.28–1.91)	0.4635
Hypercalcemia yes vs. no	8 vs. 124	27.6 (4.4–31.2) vs. 28.6 (20.2–37.4)	0.57 (0.18–1.83)	0.2153
High LDH * yes vs. no	12 vs. 120	7.9 (3.1–26.6) vs. 31.2 (21.0–37.4)	0.34 (0.11–1.10)	0.0024
HALP Low ** vs. high	64 vs. 68	17.4 (10.0–21.4) vs. 33.0 (26.6–42.9)	0.56 (0.35–0.89)	0.0114
PFS0 < 12 m. yes vs. no	86 vs. 46	25.9 (18.4–33.0) vs. 32.2 (20.1–42.9)	0.82 (0.51–1.31)	0.4119
Nivolumab third line yes vs. no	52 vs. 80	26.6 (14.8–32.2) vs. 31.8 (20.7–42.7)	0.87 (0.54–1.38)	0.5290

Abbreviations: N, number of patients; ECOG, Eastern Cooperative Oncology Group; ccRCC, clear-cell renal cell carcinoma; LDH, lactate dehydrogenase; HALP, a score combining pretreatment hemoglobin, albumin, lymphocytes, and platelets; PFS0: progression-free survival of previous line of treatment in months (m.); mOS, median overall survival; CI, confidence interval; HR, hazard ratio; * more than 1.5 times the upper limit; ** HALP ≤ 27.53 was considered low.

**Table 9:** Overall survival by risk factors in the metastatic ccRCC population (multivariate analysis).

Variables	*p*
Poor performance status (ECOG 2)	<0.0001
High LDH (more than 1.5-times of upper limit)	0.0001
HALP ≤ 27.53	0.0313

Abbreviations: ECOG, Eastern Cooperative Oncology Group; LDH, lactate dehydrogenase; HALP, a score combining pretreatment hemoglobin, albumin, lymphocytes, and platelets.

## Data Availability

The original contributions presented in this study are included in the article. Further inquiries can be directed to the corresponding author.
